# Leveraging Population Health Expertise to Enhance Community Benefit

**DOI:** 10.3389/fpubh.2020.00088

**Published:** 2020-03-31

**Authors:** Sue A. Kaplan, Marc N. Gourevitch

**Affiliations:** Department of Population Health, NYU Grossman School of Medicine, NYU Langone Health, New York, NY, United States

**Keywords:** hospital community benefit, health systems and community partnerships, hospitals addressing social determinants of health, community health improvement plan, departments of population health

## Abstract

As the Internal Revenue Service strengthens the public health focus of community benefit regulations, and many states do the same with their tax codes, hospitals are being asked to look beyond patients in their delivery system to understand and address the needs of geographic areas. With the opportunities this affords come challenges to be addressed. The regulations' focus on population health is not limited to a defined clinical population—and the resulting emphasis on upstream determinants of health and community engagement is unfamiliar territory for many healthcare systems. At the same time, for many community residents and community-based organizations, large medical institutions can feel complicated to engage with or unwelcoming. And for neighborhoods that have experienced chronic underinvestment in upstream determinants of health—such as social services, housing and education—funds made available by hospitals through their community health improvement activities may seem insufficient and unreliable. Despite these regulatory requirements, many hospitals, focused as they are on managing patients in their delivery system, have not yet invested significantly in community health improvement. Moreover, although there are important exceptions, community health improvement projects have often lacked a strong evidence base, and true health system-community collaborations are relatively uncommon. This article describes how a large academic medical center tapped into the expertise of its population health research faculty to partner with local community-based organizations to oversee the community health needs assessment and to design, implement and evaluate a set of geographically based community-engaged health improvement projects. The resulting program offers a paradigm for health system investment in area-wide population health improvement.

## Introduction

On March 23, 2010, the Patient Protection and Affordable Care Act (ACA) added a new section 501(r) to the Internal Revenue Code creating “Additional Requirements for Charitable Hospitals” (1). Pursuant to these provisions, not-for-profit hospitals are required to undertake a community health needs assessment (CHNA) every 3 years and then develop an implementation strategy—a set of “community health improvement” activities—to address priorities that are identified through that process (2). A number of states have similar policies in their tax codes. For example, the New York State Department of Health for many years has mandated that every not-for-profit hospital submit a Community Service Plan (CSP) to the State. Beginning with the CSPs that were due in the fall of 2013, the State sharpened its public health focus, requiring hospitals to align their plans with local health department priorities, which, in turn, were to align with the State's “Prevention Agenda” (3).

These federal and state regulations have been designed to: (a) open healthcare systems to greater community input; (b) foster “greater collaboration between state and local health agencies and hospitals serving the region;” (4) and (c) leverage hospital resources to advance area population health (3). Yet effective implementation of these requirements is typically challenging both for hospitals and for the community organizations with which they seek to partner. For many health care systems, focused as they are on the complexities of managing care within their walls, engaging with community partners and developing programs to improve population health call upon unfamiliar skills (5). At the same time, for many community residents and community-based organizations, large medical institutions can feel bewildering or unwelcoming. And for neighborhoods that have experienced chronic underinvestment in the upstream determinants of health—social services, housing, and education—the funds made available by hospitals through these community health improvement activities may seem insufficient and unreliable.

Community health improvement resources are one of the myriad assets that healthcare systems have —as clinical providers, employers, educational institutions, purchasers, and investors—that can be leveraged to strengthen the drivers of health in the communities in which they are located (6). Over the past few years, innovative health systems have begun to recognize these levers and look upstream to address social determinants of health—whether out of a sense of mission, to be in compliance with state regulations, to enhance reputation, to attract and maintain staff and patients, or to prepare for anticipated changes in reimbursement (7). Examples are beginning to emerge for how these efforts can be structured and sustained (8–10).

Based on the experience of one major academic health system—New York University Langone Health (NYULH)—we describe a model of how population health expertise can be brought to bear to address community health improvement requirements as part of a community-engaged approach that results in sustainable improvements in population health.

## Defining and Engaging Community and Setting Priorities

For academic medical centers, particularly those located in cities dense with other healthcare systems, defining a “community” can present a challenge. NYULH serves a broad geographic area: its primary service area includes the New York City boroughs of Manhattan, Brooklyn, and Queens, and its secondary service area extends into the borough of Staten Island, as well as Long Island, Westchester, and New Jersey. To enhance the impact of the CSP and create opportunities for synergy across programs, NYULH in 2013 narrowed the geographic scope of its CSP (previously the entire lower third of Manhattan) to focus on the closest areas of greatest need: the Lower East Side and Chinatown (together comprising Manhattan Community District 3). Following merger in 2017 with a community hospital (Lutheran Medical Center) and associated network of Federally Qualified Health Centers in Brooklyn, the CSP extended into the Sunset Park neighborhood of Brooklyn.

The three neighborhoods comprising NYULH's CSP catchment area—the Lower East Side, Chinatown and Sunset Park—share many characteristics and face similar challenges. Each is a microcosm for the social, economic, and linguistic diversity of New York City and has served as a first destination for immigrants, with high percentages of residents who are foreign born and with large Latino and Asians populations. Even as these neighborhoods gentrify, residents continue to experience high levels of poverty, low educational attainment, and health disparities.

At the same time, each neighborhood benefits from strong networks of community-based organizations (CBOs) that provide services and support for residents. Information about health status and trends in these communities, as well as our process for assessing assets and needs and setting priorities, can be found in our comprehensive Community Health Needs Assessment and Implementation Plan at https://nyulangone.org/files/chna-csp-final-8-5-19-complete-1.pdf.

Aligning with the New York State and New York City public health and community priorities, the NYULH Community Service Plan engages multiple sectors (e.g., healthcare, education, social service, faith-based organizations, and housing providers) in its goals of: (a) preventing chronic diseases by reducing tobacco use and preventing and addressing obesity, and (b) promoting healthy women, infants and children through programs focusing on parenting and teen health. These goals were selected based upon the CHNA we conducted, which analyzed and presented to the community primary and secondary data about community needs and priorities in Manhattan Community District 3 and in Sunset Park, including data from the New York City Department of Health and Mental Hygiene's Community Health Survey and the New York City Department of City Planning, as well as focus groups, surveys, interviews and meetings with residents and other community stakeholders. The priorities selected reflect continued community concern about ongoing health disparities, including tobacco use, obesity, early childhood development, and teen health. In addition, the connection between housing quality/security and health emerged as a growing concern, which led to the formation of the Brooklyn Health and Housing Consortium described below.

To oversee the need and asset assessments, priority setting, and implementation of the CSP, we formed a Coordinating Council led by the Department of Population Health and composed of NYULH faculty and staff, leadership and staff of partnering CBOs, community leaders (including community health workers, faith-based leaders, Community Board members), and a growing group of other stakeholders including researchers and policymakers. Beginning in 2017, we fully integrated partners from the NYULH Brooklyn-based system, including its affiliated network of Federally Qualified Health Centers, the Family Health Centers at NYU Langone, which now co-leads the group.

Each CSP initiative has at least one faculty partner and one community partner. To enable full participation of community partners, we have sought to ensure that the CSP program budgets cover not only the time of CBO staff who work directly on the project but also a portion of senior management time, recognizing the importance of their supervisory roles and their participation as leaders on the Coordinating Council. As one community partner observed, in partnering with academic institutions, senior staff of community organizations are often asked to contribute their time *pro bono*, straining already tight budgets.

The Coordinating Council serves as the forum for coordinating across the CSP initiatives, identifying shared challenges and emerging community needs, and grounding the work in a community based participatory approach (CBPA). In the first year of the CSP, we reviewed principles of community engagement and sought to anticipate potential causes of tension (11). From our previous experience in community based participatory research (12–14), and from early conversations with key informants as part of the CHNA, we were acutely aware of the potential for misunderstanding between academic institutions and community partners. A small group of faculty and community leaders drafted a memorandum of understanding, which provided detailed language about collaboration in program development and implementation, data sharing, and the development of presentations and publications, including the expectation of co-authorship. More recently, growing out of two CBPA projects (an assessment of the health needs and priorities of the Arab American community in southwest Brooklyn and an asset and needs assessment of Red Hook, a neighboring community in Brooklyn) the Coordinating Council revisited and revised its guiding CBPA principles and is in the process of identifying the capacity building activities and skills that are needed to support the movement of our projects further along the spectrum of community engagement (15). The principles, which grew out of a review of the extensive literature on CBPA and academic-community partnerships (16–23), are currently being reviewed and revised by our community partners and with community residents, and will then will be posted and shared as a possible starting place for other community health improvement plans.

## Leveraging Population Health Expertise in Evidence-Based Programs

State and federal regulations governing community health improvement projects require that hospitals select evidence-informed interventions that meet the needs identified in the CHNA, describe their anticipated impact, and set forth a measurement and evaluation plan (2, 3, 24). To take advantage of expertise in the design, implementation and evaluation of evidence-based programs, beginning in 2012, NYULH transferred responsibility for the CHNA and the development of its CSP from its corporate office of Strategy, Planning and Business Development to its academic Department of Population Health.

In developing an initial portfolio of community health improvement projects, faculty with population health expertise drew upon existing grant-funded evidence-informed programs designed to address the health needs of underserved populations, primarily low-income Latinx and African Americans. Building on this foundation, faculty partnered with community-based organizations to adapt those programs, tools and materials for implementation in their settings and to reflect the needs and preferences of their diverse populations, leveraging, and enriching faculty's understanding of cultural and linguistic translation, behavior change, and implementation science. The following two examples illustrate this process (A fuller picture of these and other CSP projects can be found at https://nyulangone.org/our-story/community-health-needs-assessment-service-plan).

### ParentCorps

ParentCorps, an evidence-based program developed by NYULH's Center for Early Childhood Health and Development, is designed to buffer the adverse effects of poverty and related stressors on early child development by engaging and supporting both parents and teachers at children's transition to school. The program is implemented in early childhood education or childcare settings and includes professional development for teachers and other caregivers and a 14-session weekly group educational series for parents and children. Two federally-funded, randomized controlled trials with more than 1,200 poor, minority children found that ParentCorps results in more supportive and nurturing home and early childhood classroom environments, higher kindergarten achievement scores (reading, writing, and math) and, among the highest-risk children, lower rates of obesity, and mental health problems (25). A benefit-cost analysis indicates that ParentCorps has the potential to yield cost savings of more than $2,500 per student (26).

Through the CSP, ParentCorps has partnered with University Settlement Society, a large social service agency with three early childhood sites, and with elementary schools located in the CSP catchment area, training nearly 200 teachers and teaching assistants and over 100 other professionals including mental health professionals, social workers, and administrators. In addition, ParentCorps staff have implemented seventeen 14-session series of the Parenting Program in English, and in Mandarin and Cantonese for the first time, reaching 555 families, in the process translating and adapting materials so that they are culturally tailored and acceptable to this new population. Based on earlier studies, we estimate that the program will increase parent knowledge, skills, and engagement in school; decrease the percentage of children with behavior problems; increase healthy eating and physical activity; and decrease the percentage of children who are overweight/obese.

### Tobacco Free Community

Despite the availability of safe and effective treatment for tobacco dependence, only a small proportion of smokers who try to quit each year use cessation therapies. This is particularly true among low-income adults and for non-English language speakers, contributing to growing disparities in smoking prevalence (27). The CSP navigator program is designed to address this gap, with a particular focus on Chinese American men, who have among the highest smoking rates in New York City. In partnership with Asian Americans for Equality (AAFE) and the Asian Smokers' Quitline (ASQ), experts from the Department of Population Health's Section on Tobacco, Alcohol, and Drugs are implementing a community navigator model that mirrors the patient navigator model developed, studied and implemented by the American Cancer Society (28). Results of this program have been comparable to other navigator programs (34% self-reported quit rate) and unusually, because of its roots in the community, AAFE has been able to reach many smokers who had never previously tried to quit or cut down.

## Planning for Sustainable Impact

Neither the Internal Revenue Service nor the Affordable Care Act mandate a dollar amount or percentage of operating budget that not-for-profit hospitals are required to allocate to their community health improvement projects. Indeed, it has been estimated that nationally only about five percent of community benefit dollars are allocated to community health improvement programs (29). Although a large figure when aggregated nationally, locally, the modest scope of such funds can limit their impact, particularly when viewed in the context of longstanding, unmet community needs. Fostering the sustainability of initiatives launched through community health improvement efforts is a way of extending the impact of limited funds. There are several strategies that health systems can use to advance this goal: (a) building capacity among partners and within the healthcare system; (b) integrating programs into operational flow and procedures; (c) supporting public policies that maintain initiatives and facilitate their diffusion; and (d) leveraging existing or new funding and resources that can be braided into the stream of support (30).

### Building Capacity

Academic medical centers can provide a wide array of capacity-building resources to CBOs in addition to providing direct funding for programs. For example, NYULH experts on tobacco cessation have led several in-depth training programs, reaching community health workers across the Community Service Plan partnership. Tobacco cessation experts from the medical center have also partnered with the Chinese American Medical Society to provide lectures on smoking cessation to their members for continuing medical education credit.

Through the Community Service Plan, the Family Health Centers at NYU Langone have also championed capacity building to support child development and school readiness. The ParentChild+ program (formerly known as the Parent-Child Home program), a national, evidence-based early literacy, parenting and school-readiness program, offers year-long training and support to Family Child Care (FCC) providers to promote school readiness for all children in their care. The impact of the program extends beyond the FCC environment. Parents whose children are enrolled at an FCC have reported changes in language and literacy behaviors at home, such as replacing screen time with book reading.

Academic medical centers can also support capacity-building by offering access to educational and professional development opportunities. Community partners are routinely invited to conferences to present their CSP-supported work, often with faculty co-authors; and NYULH faculty provide technical assistance and consultation on data analysis to support program evaluation and needs assessments. Recently, the CSP staff launched a series of monthly workshops inviting faculty, staff, and community experts to present on topics that reflect shared program needs and interests. The workshops have addressed survey development, in which partners were invited to bring draft instruments for review and discussion; m-health strategies in community settings; approaches to health literacy; and mindfulness for health professionals. As we have deepened our focus on CBPA, these sessions will be used to build capacity across all current, planned and future projects to do more deeply engaged community work. Emerging topics include: how to define the relevant community or communities; understanding community organizing principles and strategies; and tools and processes to promote trust, engagement, self-reflection, and equity. In addition, our quarterly Coordinating Council meetings foster cross-project learning, for example through discussion of strategies and approaches for community engagement and facilitating behavior change across cultures (31). These forums also provide an opportunity for CBOs to network with other organizations and with policymakers and potential funders.

As others have noted, the CHNA provides an opportunity for “community-engaged, health equity research” (32). Indeed, in partnering with community-based organizations, it is important for hospitals to recognize that, done right, capacity building is bidirectional. Through the discussions in our Coordinating Council, we are able organically to identify issues that have not yet emerged through more formal needs assessments or in existing data. These have included, for example, the intergenerational needs of Chinese American families in which children are raised abroad in their early years (33), and very early on we learned of the growing concern among undocumented immigrants who fear seeking care and accessing entitlements. These insights have generated new program approaches and opportunities for timely and important responses and research. In addition, partnerships can provide an opportunity to collect pilot data to support collaborative grant development. For example, as part of our Tobacco Free Community initiative, we conducted focus groups with residents in public housing about their attitudes toward the federally-mandated smoking ban being implemented in their apartment buildings, providing helpful information to the New York City Housing Authority as it rolls out its program and serving as pilot data for a large collaborative study (funded by the National Institutes of Health) of the impact of this new regulation.

Relationships with partners can also provide educational opportunities, including site visits for medical students and student research projects. Finally, an unexpected consequence of the Coordinating Council structure has been that it has facilitated productive relationships across divisions within the Department of Population Health, across departments within the medical center, and with other schools across the university.

### Integrating Programs Into Operational Flow

Programs are more likely to be sustained if they are aligned with organizational culture and priorities and integrated into operational flow and standard operating procedures (34). For this reason, in implementing the Greenlight program, a practice-based obesity prevention program, we worked closely with colleagues at the Charles B. Wang Community Health Center to minimize burden on health care providers and to integrate the program into the flow of the busy pediatric practice of this Federally Qualified Health Center. This has meant collaboratively designing program implementation with administrators to take advantage of patient waiting times and working with existing staff who provide materials and coaching. The successes and core insights from the Manhattan implementation are being used to align the program with the pediatric workflow in the Seventh Avenue Family Health Center site in Sunset Park.

Similarly, AAFE now screens for tobacco use on all of its intake forms (for example, for housing, insurance, small business development) and provides information about smoking cessation at community meetings on a wide array of topics, having learned that people are more amenable to hearing about tobacco cessation when other services are being provided and other problems solved.

### Promoting Policy Change and Program Diffusion

Engaging policymakers has been a core strategy of the Tobacco Free Community initiative. Growing out of and supported by the CSP partnership and the RCHN Community Health Foundation, the Charles B. Wang Community Health Center spearheaded the creation of a City-wide anti-smoking coalition, which helped field a street intercept survey in Chinese American neighborhoods, testified before the City Council, and worked with the New York City Department of Health and Mental Hygiene in developing and publicizing an *Epi Data Brief* that highlights cancer as the leading cause of death for Chinese New Yorkers, reflecting the persistently high rates of smoking among Asian American men (35). In response, the City Health Department launched an Asian language public awareness campaign. One of the Coalition partners, Korean Community Services, received funding from the City Council to support a tobacco navigator program in the Korean American Community, and the effort is now being expanded to include other immigrant-serving CBOs.

### Leveraging Resources

Although the scale of community health improvement funding alone is insufficient to support sustainable and long-term change, these dollars can be used to leverage other resources. Some have suggested creating pooled “community health trusts” that might attract broader investment (36). Others have used community health improvement dollars to “unlock” capital investments (37). At a programmatic level, we have sought to pool support by linking to a wide range of resources. For example, the smoking cessation program uses existing relationships and forums to direct people to available resources: the New York State Smokers' Quitline and to the Asian Smokers' Quitline, both of which offer free coaching and nicotine replacement therapy. In addition, the Robin Hood Foundation provided substantial supplemental funding for the CSP Health + Housing Initiative, a pilot housing-based community health worker project in two affordable buildings on the Lower East Side (38). The initiative is now being sustained and expanded in two additional buildings by the owners of one of the buildings in which it was piloted, in continued collaboration with our community partner, Henry Street Settlement. This not only provides a potentially sustainable and replicable funding source, but it also gives our partners ownership over the initiative and allows them to tailor the program to meet ongoing needs.

Similarly, the Family Health Centers' Project SAFE, a peer education program employing an evidence-based youth development approach to prevent teen pregnancy and HIV/AIDS, was able to deepen their reach in schools through the Community Service Plan, which was then leveraged to acquire federal Substance Abuse and Mental Health Services Administration grant funding. Leveraging community health improvement funding to access outside support not only increases the pool of available dollars, but also helps to increase visibility and demonstrate program value to internal and external audiences.

## Challenges and Lessons Learned

In launching the CSP, we experienced a number of challenges. Within our own institution, there were tensions as the Department of Population Health applied a more rigorous set of criteria to the programs that would be funded through the plan. This meant eliminating some projects that had deep institutional roots but lacked a strong evidence base or were more focused on data collection and research than on service delivery. In addition, as noted above, we brought to the CSP a set of expectations about community engagement that differed from the traditional academic approach. We have found, however, that faculty and staff have relished the deep community relationships and the egalitarian nature of the Coordinating Council, which brings together community health workers and senior faculty, policymakers and staff (31).

Developing trusting relationships with community partners presented another challenge. The CHNA regulations are specifically designed to require that hospitals open their doors to community input. Our initial foray into the community was revelatory—and sometimes painful. Overtures to some prospective partners were met with a high degree of skepticism. Several were critical of the medical center and the university's role in the community, noting a previous lack of engagement. Significant time was spent assuring community leaders of our commitment to true partnership. Fostering a strong community-based culture and identity within the Coordinating Council has been critically important to maintaining credibility with our partners and in the communities in which we are working.

The challenge of matching evidence-based community-oriented programs with community priorities has meant that our work is held together more by a set of principles and an approach than by a defined goal or outcome. Although each project has an evaluation component, “moving the needle” at a population level remains an elusive goal. This is complicated further by our geographic spread, spanning several diverse communities. A more laser-like focus on an issue or geographic area might have aligned our projects toward a single measurable outcome. But our approach has helped build the partnership and has allowed us to be responsive to needs and to generate new and promising initiatives as opportunities arise. For example, growing out of our work and deep community engagement, we have developed the Brooklyn Health and Housing Consortium, which engages health care providers, CBOs, and housing providers with the goal of developing relationships and infrastructure, and building capacity to support people with complex health and housing needs. Similarly, we have created a Community Health Worker Research and Resource Center to serve as a resource to CBOs, health systems, municipal agencies, and research organizations that are planning, or seeking to strengthen, initiatives that use lay health workers to enhance care, link services, and improve community health. These more recent efforts are evidence of a deeper level of engagement and lasting contribution to local health improvement capacity. The value of these initiatives would not likely be captured in a traditional cost-benefit approach.

## Conclusion

Community health improvement funding provides an important resource to support community-based population health initiatives. But the absence of a required funding threshold and general lack of hospital expertise in partnering to address the upstream determinants of health, threaten to limit its impact. Despite the ACA requirement for a thorough needs assessment and implementation plan, and similar mandates in many state tax codes, many hospitals have not invested deeply in community health improvement. Moreover, although there are important exceptions, community health improvement projects have often lacked a strong evidence base, and true community collaborations are difficult to achieve and sustain (39).

As hospitals begin to develop departments of population health (40), they can leverage that growing expertise—in data collection and analysis, in implementation science, in partnering to promote health and wellness outside their walls—to guide their community health improvement programs and widen the lens from patients in the delivery system to residents in the community. In this way, community benefit resources can be deployed more effectively to address important community health priorities, build community and institutional capacity, and lay a foundation for long-term sustainable change.

## Data Availability Statement

The raw data supporting the conclusions of this article will be made available by the authors, without undue reservation, to any qualified researcher.

## Author Contributions

SK and MG contributed to the design and implementation of the programs described and to the writing of the manuscript.

### Conflict of Interest

The authors declare that the research was conducted in the absence of any commercial or financial relationships that could be construed as a potential conflict of interest.
